# The Acute Optic Neuritis Network (ACON): Study protocol of a non-interventional prospective multicenter study on diagnosis and treatment of acute optic neuritis

**DOI:** 10.3389/fneur.2023.1102353

**Published:** 2023-02-24

**Authors:** Susanna Asseyer, Nasrin Asgari, Jeffrey Bennett, Omer Bialer, Yolanda Blanco, Francesca Bosello, Anna Camos-Carreras, Edgar Carnero Contentti, Sara Carta, John Chen, Claudia Chien, Mashina Chomba, Russell C. Dale, Josep Dalmau, Kristina Feldmann, Eoin P. Flanagan, Caroline Froment Tilikete, Carolina Garcia-Alfonso, Joachim Havla, Mark Hellmann, Ho Jin Kim, Philipp Klyscz, Frank Konietschke, Chiara La Morgia, Marco Lana-Peixoto, Maria Isabel Leite, Netta Levin, Michael Levy, Sara Llufriu, Pablo Lopez, Itay Lotan, Alessandra Lugaresi, Romain Marignier, Sara Mariotto, Susan P. Mollan, Cassandra Ocampo, Frederike Cosima Oertel, Maja Olszewska, Jacqueline Palace, Lekha Pandit, José Luis Peralta Uribe, Sean Pittock, Sudarshini Ramanathan, Natthapon Rattanathamsakul, Albert Saiz, Sara Samadzadeh, Bernardo Sanchez-Dalmau, Deanna Saylor, Michael Scheel, Tanja Schmitz-Hübsch, Jemal Shifa, Sasitorn Siritho, Pia S. Sperber, Prem S. Subramanian, Alon Tiosano, Adi Vaknin-Dembinsky, Alvaro Jose Mejia Vergara, Adi Wilf-Yarkoni, Luis Alfonso Zarco, Hanna G. Zimmermann, Friedemann Paul, Hadas Stiebel-Kalish

**Affiliations:** ^1^Experimental and Clinical Research Center, A Cooperation Between the Max Delbrück Center for Molecular Medicine in the Helmholtz Association and Charité Universitätsmedizin Berlin, Berlin, Germany; ^2^Charité – Universitätsmedizin Berlin, Corporate Member of Freie Universität Berlin and Humboldt-Universität zu Berlin, Experimental and Clinical Research Center, Berlin, Germany; ^3^Max Delbrück Center for Molecular Medicine in the Helmholtz Association (MDC), Berlin, Germany; ^4^NeuroCure Clinical Research Center, Charité – Universitätsmedizin Berlin, Corporate Member of Freie Universität Berlin and Humboldt-Universität zu Berlin, Berlin, Germany; ^5^Department of Neurology, Slagelse Hospital, Slagelse, Denmark; ^6^Institutes of Regional Health Research and Molecular Medicine, University of Southern Denmark, Odense, Denmark; ^7^Programs in Neuroscience and Immunology, Departments of Neurology and Ophthalmology, Sue Anschutz-Rodgers Eye Center, University of Colorado Anschutz Medical Campus, Aurora, CO, United States; ^8^Department of Neuro-Ophthalmology, Rabin Medical Center, Petah Tikva, Israel; ^9^Sackler Faculty of Medicine, Tel Aviv University, Tel Aviv, Israel; ^10^Neuroimmunology and Multiple Sclerosis Unit, Neurology Service, Hospital Clinic de Barcelona, and Institut d'Investigacions August Pi i Sunyer (IDIVAPS), University of Barcelona, Barcelona, Spain; ^11^Neurology Unit, Department of Neurosciences, Biomedicine, and Movement Sciences, University of Verona, Verona, Italy; ^12^Ophthalmology Department, Hospital Clínic de Barcelona, Institut d'Investigacions Biomèdiques August Pi i Sunyer (IDIBAPS), University of Barcelona, Barcelona, Spain; ^13^Neuroimmunology Unit, Department of Neuroscience, Hospital Aleman, Buenos Aires, Argentina; ^14^Department of Ophthalmology and Neurology, Mayo Clinic, Rochester, MN, United States; ^15^Department of Internal Medicine, University Teaching Hospital, Lusaka, Zambia; ^16^Clinical Neuroimmunology Group, Kids Neuroscience Centre, Sydney, NSW, Australia; ^17^Faculty of Medicine and Health and Brain and Mind Centre, University of Sydney, Sydney, NSW, Australia; ^18^TY Nelson Department of Paediatric Neurology, Children's Hospital Westmead, Sydney, NSW, Australia; ^19^ICREA-IDIBAPS, Service of Neurology, Hospital Clínic, University of Barcelona, Barcelona, Spain; ^20^Department of Neurology, University of Pennsylvania, Philadelphia, PA, United States; ^21^Laboratory Medicine and Pathology, Departments of Neurology, Center for MS and Autoimmune Neurology, Mayo Clinic, Rochester, MN, United States; ^22^Neuro-Ophthalmology Unit, Pierre Wertheimer Neurological Hospital, Hospices Civils de Lyon, Lyon 1 University, Lyon Neuroscience Research Center, INSERM U1028, CNRS UMR5292, IMPACT Team, Lyon, France; ^23^Pontificia Universidad Javeriana and Hospital Unviersitario San Ignacio, Bogotá, Colombia; ^24^Institute of Clinical Neuroimmunology, LMU Hospital, Ludwig-Maximilians-Universität München, Munich, Germany; ^25^Department of Neurology, National Cancer Center, Goyang, Republic of Korea; ^26^Neurology Unit, IRCCS Institute of Neurological Sciences, Bologna, Italy; ^27^Department of Biomedical and Neuromotor Sciences, University of Bologna, Bologna, Italy; ^28^CIEM MS Center, Federal University of Minas Gerais Medical School, Belo Horizonte, Brazil; ^29^Department of Neurology, Oxford University Hospitals, National Health Service Trust, Oxford, United Kingdom; ^30^Department of Neurology, Hadassah Medical Center, Hebrew University, Jerusalem, Israel; ^31^Neuromyelitis Optica Research Laboratory, Massachusetts General Hospital and Harvard Medical School, Boston, MA, United States; ^32^Neuroimmunology and Multiple Sclerosis Unit, Neurology Service, Hospital Clinic de Barcelona, Barcelona, Spain; ^33^Institut d'Investigacions August Pi i Sunyer (IDIVAPS), University of Barcelona, Barcelona, Spain; ^34^IRCCS Istituto delle Scienze Neurologiche di Bologna, Bologna, Italy; ^35^Dipartimento di Scienze Biomediche e Neuromotorie, Università di Bologna, Bologna, Italy; ^36^Birmingham Neuro-Ophthalmology, Queen Elizabeth Hospital, University Hospitals Birmingham NHS Foundation Trust, Birmingham, United Kingdom; ^37^Translational Brian Science, Institute of Metabolism and Systems Research, University of Birmingham, Edgbaston, United Kingdom; ^38^Faculty of Medicine, University of Botswana, Gaborone, Botswana; ^39^Center for Advanced Neurological Research, KS Hegde Medical Academy, Nitte (Deemed to be University), Mangalore, India; ^40^Translational Neuroimmunology Group, Kids Neuroscience Centre, Children's Hospital Westmead, Sydney, NSW, Australia; ^41^Department of Neurology, Concord Hospital, Sydney, NSW, Australia; ^42^Siriraj Neuroimmunology Center, Faculty of Medicine Siriraj Hospital, Mahidol University, Bangkok, Thailand; ^43^Department of Neurology, Johns Hopkins University School of Medicine, Baltimore, MD, United States; ^44^Department of Neuroradiology, Charité – Universitätsmedizin Berlin, Corporate Member of Freie Universität Berlin and Humboldt-Universität zu Berlin, Berlin, Germany; ^45^Department of Surgery, University of Botswana, Gaborone, Botswana; ^46^Neuroscience Center, Bumrungrad International Hospital, Bangkok, Thailand; ^47^German Center for Cardiovascular Research (DZHK), Berlin, Germany; ^48^Department of Ophthalmology, Fundación Universitaria Sanitas Facultad de Medicina, Bogotá, Colombia; ^49^Department of Neurology, Sackler Faculty of Medicine, Tel-Aviv University, Tel-Aviv, Israel; ^50^Einstein Center Digital Future, Berlin, Germany

**Keywords:** Aquaporin-4-IgG (AQP4-IgG), clinically isolated syndrome (CIS), MOG-IgG associated disorders (MOGAD), multiple sclerosis (MS), neuromyelitis optica spectrum disorders (NMOSD), optic neuritis (ON)

## Abstract

Optic neuritis (ON) often occurs at the presentation of multiple sclerosis (MS), neuromyelitis optica spectrum disorders (NMOSD), and myelin oligodendrocyte glycoprotein (MOG) antibody-associated disease (MOGAD). The recommended treatment of high-dose corticosteroids for ON is based on a North American study population, which did not address treatment timing or antibody serostatus. The Acute Optic Neuritis Network (ACON) presents a global, prospective, observational study protocol primarily designed to investigate the effect of time to high-dose corticosteroid treatment on 6-month visual outcomes in ON. Patients presenting within 30 days of the inaugural ON will be enrolled. For the primary analysis, patients will subsequently be assigned into the MS-ON group, the aquapotin-4-IgG positive ON (AQP4-IgG+ON) group or the MOG-IgG positive ON (MOG-IgG+ON) group and then further sub-stratified according to the number of days from the onset of visual loss to high-dose corticosteroids (*days-to-Rx*). The primary outcome measure will be high-contrast best-corrected visual acuity (HC-BCVA) at 6 months. In addition, multimodal data will be collected in subjects with any ON (CIS-ON, MS-ON, AQP4-IgG+ON or MOG-IgG+ON, and seronegative non-MS-ON), excluding infectious and granulomatous ON. Secondary outcomes include low-contrast best-corrected visual acuity (LC-BCVA), optical coherence tomography (OCT), magnetic resonance imaging (MRI) measurements, serum and cerebrospinal fluid (CSF) biomarkers (AQP4-IgG and MOG-IgG levels, neurofilament, and glial fibrillary protein), and patient reported outcome measures (headache, visual function in daily routine, depression, and quality of life questionnaires) at presentation at 6-month and 12-month follow-up visits. Data will be collected from 28 academic hospitals from Africa, Asia, the Middle East, Europe, North America, South America, and Australia. Planned recruitment consists of 100 MS-ON, 50 AQP4-IgG+ON, and 50 MOG-IgG+ON. This prospective, multimodal data collection will assess the potential value of early high-dose corticosteroid treatment, investigate the interrelations between functional impairments and structural changes, and evaluate the diagnostic yield of laboratory biomarkers. This analysis has the ability to substantially improve treatment strategies and the accuracy of diagnostic stratification in acute demyelinating ON.

**Trial registration:**

ClinicalTrials.gov, identifier: NCT05605951.

## Introduction

Optic neuritis (ON) is the most common optic neuropathy in young adults with an annual incidence rate of three to five per 100,000 person-years (1–4). ON involves primary inflammation, demyelination, and axonal injury in the optic nerves and the chiasm (1, 5). This can lead to retinal ganglion cell destruction and significant visual loss (4, 6). ON can be the initial event in multiple sclerosis (MS), including clinically isolated syndrome (CIS) (3), in aquaporin-4-IgG positive (AQP4-IgG+) and seronegative neuromyelitis optica spectrum disorders (NMOSD) (7), and in myelin oligodendrocyte glycoprotein-IgG (MOG-IgG+)-associated disease (MOGAD) (8). The incidence of ON is stable and similar around the world (4, 9), but the proportion of ON patients with AQP4-IgG+ON and MOG-IgG+ON vs. MS-ON differs greatly in different races (4). Visual outcomes vary between the three diseases (10): in MS-ON and MOG-IgG+ON, visual prognosis is good (10–13), but AQP4-IgG+ON is associated with significant visual loss (11, 14–16). As the three disease entities require different acute and long-term treatment strategies, earlier diagnostic stratification has the potential to assist tailored treatment decisions and thereby improve visual outcomes (17).

**Figure 1:**
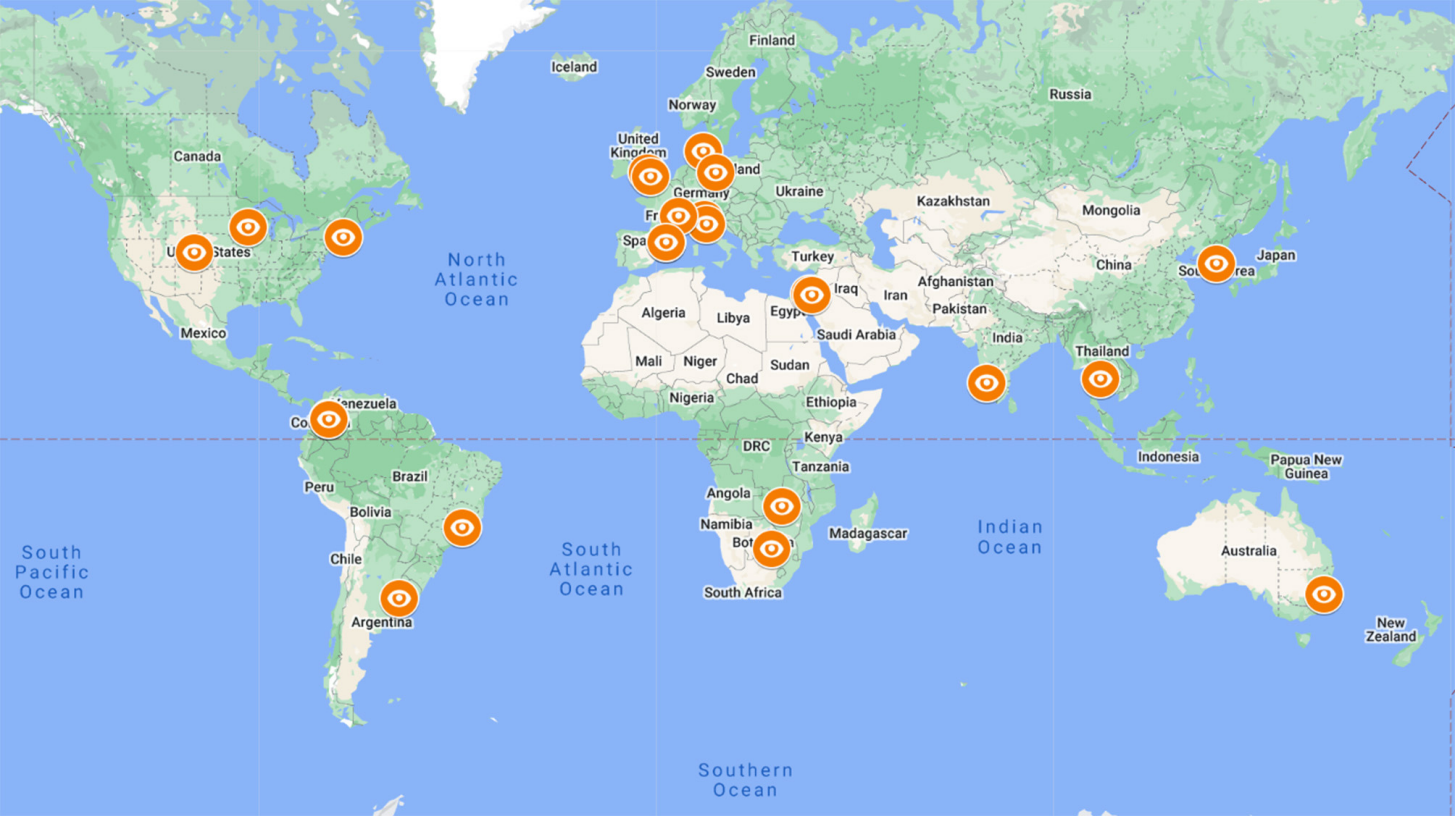
Participating ACON centers. Twenty-eight ACON centers (status 10/2022) Africa (Botswana, Zambia), Asia (India, Korea, Thailand, Vietnam), the Middle East (Israel), North America (USA), South America (Argentina, Brazil, Colombia), Australia, and Europe (Denmark, France, Germany, Italy, Spain, United Kingdom).

The Acute Optic Neuritis Network (ACON) is a global cooperation of 28 academic centers longitudinally investigating subjects with inaugural acute ON (Figure 1). Here, we present the protocol for the ACON study, which primarily aims to evaluate the effect of time to corticosteroid treatment (measured as the number of days from onset of visual loss to treatment with high-dose corticosteroids; *days-to-Rx*) on visual and structural outcomes in MS-ON, AQP4-IgG+ON, and MOG-IgG+ON respecting the novel diagnostic criteria for ON (18).

### The effect of hyperacute high-dose corticosteroid treatment on functional outcome in inaugural MS-ON, AQP4-IgG+ON, and MOG-IgG+ON

Current treatment protocols for ON are based on the landmark 1992 Optic Neuritis Treatment Trial (ONTT), which involved 457 patients with new onset ON. The trial included three arms: placebo, 250 mg of IVMP every 6 hours for three days followed by oral prednisone at 1 mg/kg for 11 days, and oral prednisone at 1 mg/kg for 14 days (19). As treatment with IVMP was associated with a more rapid recovery of visual function and showed the most benefit within the first 15 days of follow-up, many centers used this protocol to treat people who present with ON. Importantly, the inclusion criteria stipulated that people with visual loss in the preceding 8 days could be enrolled in the ONTT (19), and the subsequent results of the ONTT showed that there was a delay in initiation of the treatment (mean 5 days ± 1.6 days) (19, 20). The ONTT was not designed to investigate the impact of time from visual loss or indeed the preceding pain to high-dose corticosteroid therapy. The ONTT was conducted prior to the advent of the serological tests for AQP4-IgG and MOG-IgG. On subsequent testing of all the ONTT participants, it did not contain any patients with AQP4-IgG+ON and only a few patients with MOG-IgG+ON (21). The recommendations formed by this landmark article are limited by the inclusion criteria and are likely of limited applicability in countries and across races where the proportion of AQP4-IgG+ON and MOG-IgG+ON is higher, as compared with North America. Preliminary evidence from several retrospective studies suggests a benefit to visual outcomes of initiating early high-dose corticosteroids to treat AQP4-IgG+ON and MOG-IgG+ON (20, 22–25). We propose verifying the functional benefit of early high-dose corticosteroids in a global, multi-racial prospective study including patients with inaugural MS-ON, AQP4-IgG+ON, and MOG-IgG+ON.

### Developing data for ON escalation treatment protocols

There is currently no consensus on escalation treatment protocols for protocols for plasma exchange (PLEX) or intravenous immunoglobulines (IVIG) in the treatment of antibody-mediated ON and a lack of specific recommendations with respect to treatment duration. With no prior studies analyzing the varying patterns of ON severity, the speed of disease progression, the degree of response to high-dose corticosteroids, and the difficulty in comparing patients presenting late vs. early after the onset of visual loss, all of which make treatment escalation guidelines difficult to develop. The development of this study and the detailed database, which accurately captures ON severity and course, builds the basis for the development of treatment escalation guidelines.

### Structural biomarkers as an aid to tailor ON treatment

To explore possible means to aid hyperacute treatment decisions, the ACON study will collect data on and evaluate the potential of structural biomarkers as methods to enhance timely diagnoses. These structural biomarkers will be explored using magnetic resonance imaging (MRI) and optical coherence tomography (OCT).

MRI of the brain and the orbits is one of the most established clinical tools for investigating acute ON. The radiological features of ON differ between the three disease etiologies: optic nerve lesions in AQP4-IgG+ON and MOG-IgG+ON are more frequently bilateral and more extensive than in MS-ON (26–28). AQP4-IgG+ON typically affects posterior parts of the optic nerve and the chiasm, while MOG-IgG+ON typically affects long segments of the anterior optic nerve (10, 28). During the acute phase of ON, optic nerve lesion length has been shown to be a useful imaging biomarker, predictive of retinal neuro-axonal loss and chronic visual impairment (29). We will explore the diagnostic and clinical relevance of visual pathway lesions and their persistence following the inaugural ON.

OCT with the peripapillary retinal nerve fiber thickness (pRNFL), the macular ganglion cell layer (GCL), or the ganglion cell complex (GCC) measurements aid differentiation between acute MS-ON and MOG-IgG+ON (30). However, age and race have been reported to impact pRNFL (31). We will re-explore whether pRNFL robustly differentiates MS-ON from MOG-IgG+ON in a multi-racial population of subjects with inaugural ON. Assessment of OCT imaging parameters such as the ganglion cell analysis may allow disease stratification and detection of subclinical activity prior to the initial ON and may provide insightful longitudinal data such as rate of progression, which has the potential to correlate with disease severity (32, 33).

### Biological biomarkers

Neurofilament light chain (NfL) is a major structural component of neurons and can be detected in the serum and cerebrospinal fluid (CSF). Elevated NfL levels are an indicator of neuronal damage (34) in acute ON (35), coinciding with visual dysfunction (36) and structural retinal damage (37). However, the role of serum NfL levels and dynamics in the acute stage of ON as potential indicators for subsequent conversion to MS, NMOSD, or MOGAD remains unexplored.

Glial fibrillary acidic protein (GFAP) is the predominant intermediate filament in mature astrocytes (38, 39) and was identified in astrogliosis MS lesions (40). It is differentially elevated in the CSF and the blood in the three inflammatory diseases of interest in this study (41–43). ACON aims to collect data on NfL and GFAP in the serum and CSF of patients following acute ON and to investigate their patterns in MS-ON, AQP4-IgG+ON, MOG-IgG+ON and also in double-seronegative non-MS-ON.

The detection of serum MOG-IgG is a crucial step for correctly diagnosing MOGAD (44). Live cell-based assays (CBA) using full-length human MOG are optimal and have consistently shown a 99% specificity for typical MOGAD phenotypes (44, 45). Many centers worldwide use a commercially available cell-based assay using fixed transfected cells (Euroimmun AG, Lübeck Germany), which has excellent (98%) specificity (46). Titers of MOG-IgG can decrease to undetectable levels following an acute attack, after treatment or disease remission. Thus, potential factors affecting the duration of seropositivity will be explored using longitudinal serum samples from patients exhibiting MOG-IgG seropositivity following acute ON. The diagnostic value of CSF antibody testing remains unclear. Since AQP4-IgG and MOG-IgG are both produced extrathecally, testing CSF is currently not routinely recommended (47). However, a few cases of seronegative NMOSD have been described, where MOG-IgG was present in CSF only (48). ACON aims to clarify the role of MOG-IgG in CSF in double-seronegative non-MS-ON.

### Clinical phenotypes

Pain patterns differ between MS-ON, AQP4-IgG+ON, and MOG-IgG+ON. Typically, both MS-ON and AQP4-IgG+ON are preceded by relatively mild retrobulbar pain, which worsens with eye movement (1, 49–51). By contrast, pain in MOG-IgG+ON is typically intense (52, 53). No comparative studies to date have investigated pain scoring to distinguish MS-ON and AQP4-IgG+ON from MOG-IgG+ON.

Measurements of visual function in daily routine and quality of life (QoL) are understudied in neuroscience, though of great value to patients. Issues impacting QoL after an ON event include perceived visual dysfunction, the degree of anxiety regarding future loss of vision and further relapses, pain patterns, depression, and adjustment difficulties. Visual function in daily routine correlates with functional (e.g., visual acuity) and structural measurements (e.g., pRNFL) of visual outcome in NMOSD (16). The long-term visual function in daily routine and Qol following ON is an understudied aspect, which may be explored through a high-quality detailed database of questionnaires filled in by patients in a prospective fashion.

In summary, ACON will build a broad dataset to serve as a platform to revise and tailor acute ON treatment recommendations, improve the differential diagnoses between neuroimmunological disease entities, and identify determinants of disease progression and QoL in subjects after inaugural MS-ON, AQP4-IgG+ON, and MOG-IgG+ON.

## Methods and analysis

### Study objectives

The primary objective of ACON is to investigate whether MS-ON, AQP4-IgG+ON, and MOG-IgG+ON patients treated with early high-dose corticosteroids for visual loss have better visual outcomes than those with late treatment. Treatment with both IVMP and oral high-dose corticosteroids is currently used as the standard of care in ON and will be included. According to previous data (24), subjects will be stratified into three groups: those presenting within 3 days of the onset of visual loss, those presenting between 4 and 7 days from the onset of visual loss, and those presenting between 8 and 30 days from onset of visual loss (days-to-Rx). The stratification will be performed within each respective disease group (MS, AQP4-IgG+NMOSD, and MOGAD).

**Figure 2:**
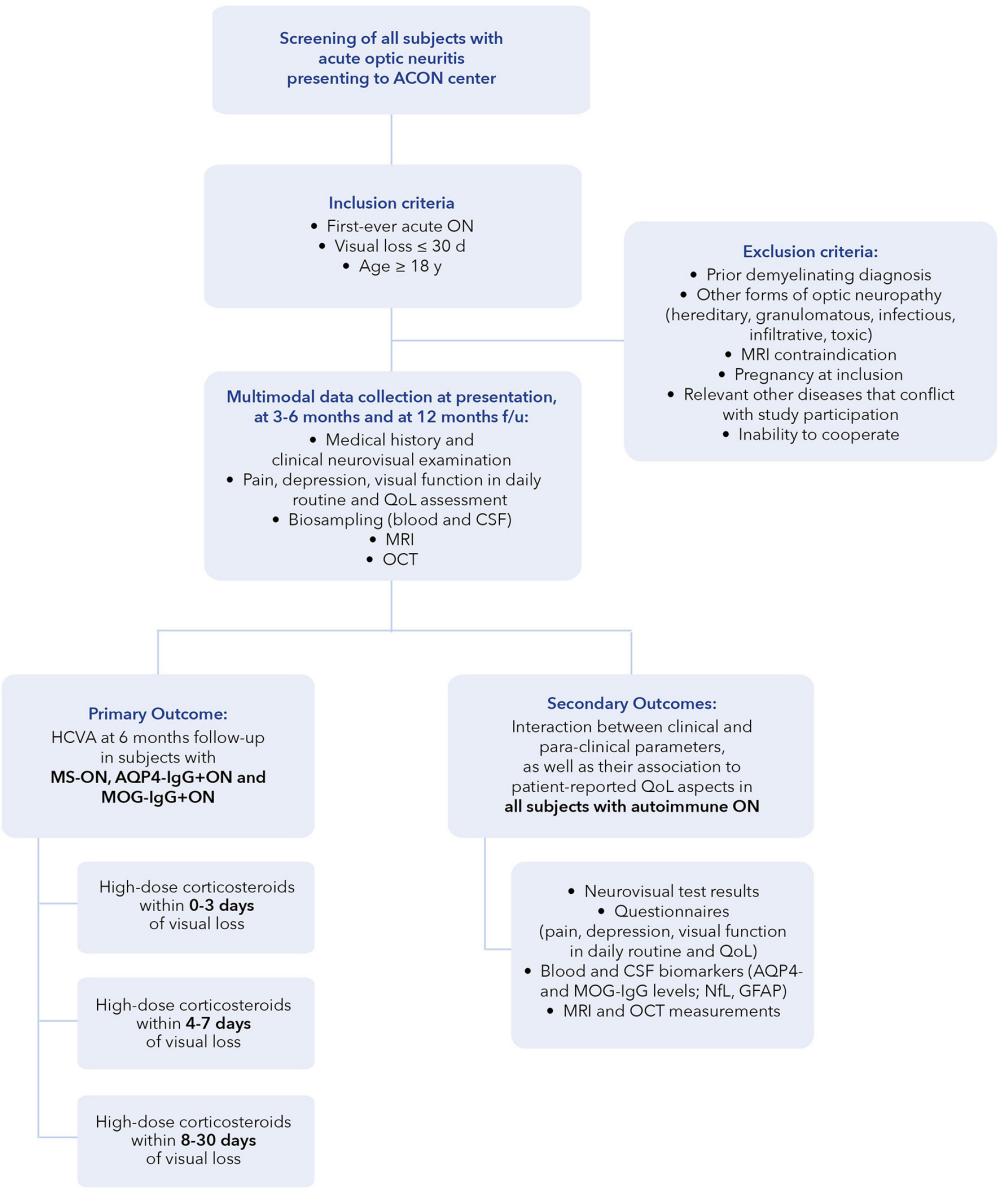
Flowchart of ACON study design. ACON, Acute Optic Neuritis Network; AQP4, aquaporin-4; CSF, cerebrospinal fluid; days; f/u, follow-up; GFAP, glial fibrillary acidic protein; MRI, magnet resonance imaging; MOG, myelin-oligodendrocyte glycoprotein; NEI-VFQ-25, National Eye Institute Visual Function Questionnaire; NfL, neurofilament light chain; OCT, optical coherence tomography; ON, optic neuritis; QoL, quality of life; y, years.

Secondary objectives will consist of the interaction between clinical and para-clinical parameters, as well as their association with patient-reported QoL aspects (see Table 1; Figure 2).

**Table 1:** Secondary objectives.

• Visual and structural outcomes of acute ON in patients treated with high-dose corticosteroid-therapy vs. plasmapheresis as first-line treatment.
• Visual and structural outcomes of MS-ON in patients treated with high-dose corticosteroid-therapy with oral prednisone taper vs. without taper as standard of care.
• Diagnostic and prognostic value of biomarker levels (NfL, GFAP) and associations with visual pathway damage (MRI- and OCT-based) in the acute stage and during follow-up.
• Characterization of MOG-IgG and AQP4-IgG levels and compartmentalisation (serum vs. CSF, using simultaneous paired samples) and associated risks for subsequent relapses in subjects with AQP4-IgG+ON and MOG-IgG+ON.
• Diagnostic value of OCT markers (e.g., increased pRNFL) for diagnosis of MS, NMOSD, and MOGAD.
• Prognostic value of OCT markers (e.g., increased pRNFL) for the visual outcome at 12-months follow-up.
• Diagnostic value of OCT markers for a conversion from acute ON to clinically definite MS.
• Diagnostic value of early clinical variables (i.e., visual loss and pain patterns).
• Investigation of the link between clinical symptoms (i.e., degree of visual loss and pain patterns) and lesion extension (detected using MRI and OCT).
• Characterization of visual function in daily routine, visual QoL scores and incidence of depression at 12-months follow-up.
• Interrelation of HC-BCVA and LC-BCVA and patient reported outcome measures at 6- and 12-months follow-up.

AQP4, aquaporin-4; CSF, cerebrospinal fluid; GFAP, glial fibrillary acidic protein; HC-BCVA, high-contrast best-corrected visual acuity; LC-BCVA, low-contrast best-corrected visual acuity; MOG, myelin-oligodendrocyte glycoprotein; MRI, magnet resonance imaging; MS, multiple sclerosis; NfL, neurofilament light chain; NMOSD, neuromyelitis optica spectrum disorders; OCT, optical coherence tomography; ON, optic neuritis; pRNFL, peripapillary retinal nerve fiber layer; QoL, quality of life.

### Study design

ACON is an international, multicenter non-interventional study network aiming to optimize treatment decisions in subjects with acute ON and to improve understanding of the underlying pathologies. It currently includes 28 teaching hospitals from Africa (Botswana, Zambia), Asia (India, Korea, Thailand, and Vietnam), the Middle East (Israel), North America (USA), South America (Argentina, Brazil, and Colombia), Australia, and Europe (Denmark, France, Germany, Italy, Spain, and United Kingdom) (for details see Supplementary material).

ACON centers will recruit and prospectively collect data from all subjects with inaugural acute ON (see study population).

Local staff will have confirmed up-to-date training in the conduct of studies according to the International Conference on Harmonization of Technical Requirements for Registration of Pharmaceuticals for Human Use (ICH) and good clinical practice (GCP) standards.

All study participants will be evaluated during hospitalization or through outpatient clinics at the time of presenting with inaugural acute ON as well as during follow-ups (at 6- and 12-month after onset). Visits at additional time points are optional, based on patient-tailored needs and the recruiting centers' standard of care. The recruitment period is planned for 36 months. Acute ON treatment is provided as part of routine care according to the clinical best practice at the respective study center. This study will not include the randomization of patients to receive early or late high-dose corticosteroids. Disease diagnosis will be assessed at 6- and 12-month follow-up visits, respecting the diagnostic criteria for MS according to the 2017 revised McDonald criteria (54), diagnosis of AQP4-IgG-positive or seronegative NMOSD according to the 2015 international consensus diagnostic criteria (55), and diagnosis of MOGAD in subjects with clinical characteristics consistent with MOGAD and positive testing of MOG-IgG with respect to the new MOGAD diagnostic criteria (44, 56, 57).

### Study population

A total of 300 patients with acute ON will be screened for study eligibility. We will include only patients with inaugural ON. Subjects presenting for the first time with isolated ON or ON with additional demyelinating syndromes, e.g., myelitis or acute disseminated encephalomyelitis (ADEM) occurring within 30 days of the acute ON, will be included. Furthermore, patients with dissemination in time and space on MRI will be included. Patients with prior soft symptoms, which can retrospectively be considered to be a demyelinating manifestation will be included, excluding patients with a prior demyelinating diagnosis or prior symptoms of optic neuritis (see exclusion criteria). The prevalence of MS-ON, AQP4-IgG+ON, and MOG-IgG+ON differs in each of the participating centers. For the primary analysis, we collect data from subjects with MS-ON, AQP4-IgG+ON, and MOG-IgG+ON. For the secondary analysis, multimodal data will be collected in subjects with any demyelinating ON (CIS-ON, MS-ON, AQP4-IgG+ON, or MOG-IgG+ON and seronegative non-MS-ON) with the aim of exploring clinical, structural, and laboratory biomarkers to expedite the diagnosis and tailoring of treatment. This data will include OCT measurements, an MRI of the visual pathway including orbital cuts, questionnaires (headache, visual function in daily routine, depression, and QoL), basal metabolic index (BMI), IVMP, and oral corticosteroid treatment duration, utility of escalation therapy with PLEX or IVIG, and serum and CSF biomarkers (AQP4- and MOG-IgG levels, NFL, and GFAP).

We expect between 30 and 50% of subjects will be ineligible due to the rigorous exclusion criteria.

The inclusion criteria include written informed consent, age ≥18 years, and diagnosis of an inaugural ON with respect to the novel diagnostic criteria for ON (18) within 30 days from the onset of visual loss and in the absence of previously diagnosed demyelinating events.

The exclusion criteria comprise other forms of optic neuropathy (e.g., glaucoma, Leber's hereditary optic neuropathy, other inherited optic neuropathies such as OPA1/OPA3-mutations, granulomatous, infectious, infiltrative, or toxic neuropathies, as well as incidental signs of prior optic neuropathy, prior events of visual loss, and clinical evidence of optic nerve thinning at presentation), other significant comorbidities (i.e., medically uncontrolled severe arterial hypertension, severe diabetes mellitus, chronic infectious diseases, drug abuse, and severe psychiatric or psychological disorders), prior demyelinating diagnosis, pregnancy at inclusion, MRI contraindications, and medical or psychological constraints impacting the ability to give informed consent to study participation and fulfill the study protocol.

The inclusion and exclusion criteria are summarized in Table 2. They are applied at the time of screening by the study physician. Throughout the study duration, we will apply drop-out criteria (see Table 2) as a guideline to evaluate the case for a premature end-of-study.

**Table 2:** Inclusion and exclusion criteria.

**Inclusion criteria**
• First-ever acute ON
• Onset of visual symptoms within maximum of 30 days
• Age ≥ 18 years
• Ability to give written informed consent
• Presence of written consent
**Exclusion criteria**
• MRI contraindication
• Prior demyelinating diagnosis
• Diagnosis of other forms of optic neuropathy (hereditary, granulomatous, infectious, infiltrative, toxic, incidental signs of prior optic neuropathy, prior events of visual loss, clinical evidence of optic nerve thinning at presentation)
• Pregnancy at inclusion
• Relevant other diseases that conflict with study participation according to protocol
• Inability to cooperate
**Drop-out criteria**
• Withdrawal of consent
• Non-compliance with the protocol (decision by study board)
• Condition hindering study continuation (decision by study board)

MRI, magnet resonance imaging; ON, optic neuritis.

### Data collection

Screening for study eligibility occurs at the time of presentation at the respective study center. Data collection according to the standard of care at the respective institution is performed in the acute phase of an inaugural acute ON (baseline visit) and at 6- and 12-month follow-up visits.

### Medical history and clinical examination

The medical history will be gathered at each visit and consists of demographics (age, sex, race), height and weight, acute attack-related symptoms with a focus on visual symptoms and pain, vaccination history, previous infections, fertility history, comorbidities, and treatment (drugs and supportive therapies).

For acute ON treatment, ACON specifies the following list of treatment options from which each center will select their choice:

(1) IVMP 1 g for 3 days followed by a taper
(2) IVMP 1 g for 5 days followed by a taper
(3) IVMP 1 g for 3 days without taper
(4) IVMP 1 g for 5 days without taper
(5) Oral prednisone 1,250 mg every other day (EOD)
(6) Others, specified.

In patients with severe visual loss (6/60 or worse) or mean visual field defect of −12 MD and worse at presentation, we recommend rechecking high-contrast best-corrected visual acuity (HC-BCVA) and visual fields on day 5 of IVMP. If vision is not improved by 2 or more lines or more than 4 DB on the visual field, we recommend to consider starting escalation treatment.

For escalation therapy, ACON proved the following treatment suggestions for patients whose vision does not improve with IVMP:

(1) PLEX for 5 days and then reassess vision
(2) Immunoadsorption
(3) IVIG at the dose of 2 g/kg over 5 days (0.4 per day).

At follow-up, all recurrent ON events as well as all demyelinating events will be captured. We will also capture and describe treatment complications and any other comorbidities occurring during the study period.

The neurological examination includes an Expanded Disability Status Scale (EDSS) according to neurostatus definitions (58, 59). The visual examination measures refraction by an autorefraction device or through direct skiascopy/retinoscopy, 100% high-contrast visual acuity, and 2.5% low-contrast visual acuity measured with Sloan charts, automated visual fields (24-2 or 30-2), and visual evoked potentials (VEP).

HC-BCVA and visual fields are the two visual parameters used globally to define blindness. In addition, HC-BCVA and visual fields are used to determine which patients have a vision that allows driving. Thus, these are two of the most clinically relevant endpoints for the patients themselves (3). Low-contrast best-corrected visual acuity (LC-BCVA) is used as a more sensitive measure of visual dysfunction (60) and will be measured in the acute setting and during follow-up.

### Pain, depression, visual function in daily routine, and QoL assessment

We will assess pain patterns using a semi-standardized interview. Questions focus on ON-related headache characteristics including the onset of pain, pain intensity, pain quality, location, duration, additional symptoms, response to pain treatment, and pain response to steroid treatment. Second, the Brief Pain Inventory (BPI) will be performed to assess (1) pain severity within the previous 24 h and (2) seven domains of pain-related interference with daily life including general activity, mood, walking ability, working ability, relations with other people, sleep, and enjoyment of life (61). The Beck's Depression Inventory version II (BDI-II) will be used to capture signs of depression both in the acute phase and during follow-ups (62). Visual quality function in daily routine is measured with the National Eye Institute Visual Function Questionnaire (NEI-VFQ-25), with its neuro-ophthalmological supplement (63). QoL is measured with the EuroQol 5-dimension (EQ-5D) index in five dimensions: mobility, self-care, usual activities, pain/discomfort, and anxiety/depression (64).

### Biosampling

Blood and CSF will be collected within one week from the presentation. Venous blood samples will be collected, comprising of serum, plasma, as well as optional PAXGen and peripheral blood mononuclear cell (PBMC) vials. Biosample analysis includes a clinical standard laboratory diagnostic panel as well as testing for AQP4- and MOG-antibodies, GFAP, and NfL. Systematic serum antibody measurements including AQP4-IgG and MOG-IgG will be performed with fixed or live cell-based assays (CBA) (65–67). MOG-IgG samples will be tested and reconfirmed employing both the commercially available assay and the human cell-based assay. Cell-based assays for MOG-IgG will either be performed at centers at which this assay is available (Mayo clinic, Barcelona, etc.) or shipped to a participating ACON center to reconfirm fixed cell-based assays.

CSF will be obtained from clinical routine diagnostics (only in the acute phase) and collected for further analysis at the respective center. Routine diagnostics include oligoclonal band detection and cell count. In addition to a clinical standard CSF analysis, MOG-IgG in CSF will be assessed. Preserves of biospecimens will be stored at −80°C for future scientific analyses.

### Magnetic resonance imaging

Cerebral MRI with dedicated orbital cuts at the baseline will be performed as part of the clinical routine diagnostic tests. Patients will receive 1.5 or 3-Tesla imaging, including a cerebral 3D T2-weighted and/or Fluid Attenuated Inversion Recovery (FLAIR) sequence, and, if available, a 3D T1-weighted Magnetization Prepared Rapid Gradient Echo (MPRAGE) sequence. In the acute setting and at 6-month follow-up, 3D T1-weighted, fat suppression (FS) sequences and/or MPRAGE will be performed following gadolinium administration. Radiological analysis parameters include brain lesion number and volume, location, and extension (for details see Supplementary material Questionnaires and Data Collection CRFs). The MRI characterization score of the optic nerve developed by Ramanathan et al. (28) takes into account optic nerve lesion extent and character, as well as the presence or absence of abnormalities in other parts of the brain.

### Optical coherence tomography

Participants will undergo OCT of the retina and the optic nerve head within 10 days from the presentation. The following Spectral Domain OCT devices will be included: Cirrus HD-OCT, Carl Zeiss Meditec, Jena, Germany; Topcon, Optovue, Canon or Spectralis, Heidelberg Engineering, Heidelberg, Germany.

Scans will be obtained in adherence to the Advised Protocol for OCT Study Terminology and Elements (APOSTEL) 2.0 nine-point recommendations and the OSCAR-IB quality criteria (68, 69). Documented OCT measurements include the average pRNFL thickness, GCL or GCC as collected by the different platforms (e.g., Spectralis measuring GCL, Zeiss measuring GCC). The OCT images will subsequently be analyzed using *post-hoc* analysis with semi-automatic, device-independent algorithms (70).

We will exclude patients with insufficient documentation or those who have their OCT imaging on time-domain devices. Low-quality spectral-domain OCT data will be excluded from the OCT analysis. We plan to qualitatively explore concomitant OCT findings, including microcystic macular oedema (MMO) or peripapillary hyperreflective ovoid mass-like structures (PHOMS) (71, 72).

Table 3 summarizes the data collection.

**Table 3:** Data collection overview.

**Collected data type**	**Study Enrollment (Screening)**	**Baseline**	**6 months follow-up**	**12 months follow-up**	**Additional study visits (optional)**
Patient demographics	X				
Informed consent	X				
Inclusion/exclusion criteria	X				
Race		X			
Semi-structured assessment of patient reported visual symptoms		X	X	X	(X)
Semi-structured pain assessment		X	X	X	(X)
Treatment history		X	X	X	(X)
Medical history and comorbidities		X	X	X	(X)
Pregnancies		X	X	X	(X)
Vaccination history		X	X	X	(X)
Relapse history		X	X	X	(X)
BPI		X	X	X	(X)
BDI-II		X	X	X	(X)
EQ-5D		(X)	(X)	(X)	(X)
Visual function in daily routine (NEI-VFQ-25 + neuro-ophthalmological supplement)		(X)	(X)	(X)	(X)
Vital signs and measurements		X	X	X	(X)
Clinical routine laboratory tests from serum and CSF, including OCB, AQP4-IgG, MOG-IgG		X	X	X	(X)
Biomarker analysis (GFAP, NfL)		X	X	X	(X)
EDSS		X	X	X	(X)
High-contrast visual acuity		X	X	X	(X)
Low-contrast visual acuity		X	X	X	(X)
Automated visual fields (24-2 or 30-2)		(X)	(X)	(X)	(X)
VEP		(X)	(X)	(X)	(X)
Cerebral MRI, including orbital cuts (lesion number, lesion volume, lesion location, number of involved segements by the ON)		X	(X)	(X)	(X)
OCT (pRNFL, macular ganglion cell analysis, morphometric markers)		X	X	X	(X)

X without brackets indicates mandatory tests, (X) indicates optional tests. AQP4, aquaporin-4; BPI, Brief Pain Inventory; CSF, cerebrospinal fluid; EDSS, Expanded Disability Status Scale; EQ-5D, EuroQol 5-Dimension EQ-5D-index; GFAP, glial fibrillary acidic protein; MRI, magnet resonance imaging; MOG, myelin-oligodendrocyte glycoprotein; NEI-VFQ-25, National Eye Institute Visual Function Questionnaire; NfL, neurofilament light chain; OCB, oligoclonal bands; OCT, optical coherence tomography; ON, optic neuritis; pRNFL, peripapillary retinal nerve fiber layer; VEP, visual evoked potentials.

### Data management

All clinical results and MRI imaging data will be pseudonymized and stored in electronic case report form (eCRF) in an electronic database hosted by Research Electronic Data Capture (REDCap), a secure web application developed by Vanderbilt University, for building and managing academic databases (73) on a secure server at Charité—Universitätsmedizin Berlin, Germany. Regular monitoring in the form of independent data quality checks is ensured. OCT image data will be analyzed at Charité—Universitätsmedizin Berlin, Germany.

### Sample size considerations

The study is exploratory, and therefore, a formal sample-size computation is not possible. Hence, we justify the sample size by feasibility, which is *n* = 100 for MS-ON and *n* = 50 for AQP4-IgG+ON and MOG-IgG+ON. For each disease (MS-ON, AQP4-IgG+ON, and MOG-IgG+ON), patients will be stratified into subgroups according to the number of days since the onset of visual loss: 0–3 days, 4–7 days, and 8–30 days until high-dose corticosteroid treatment. With *n* = 50, the width of the 95% confidence interval for the treatment effects (=difference between visual acuities between these strata) is ~0.55 standard deviations of the mean difference depending on the actual number of patients within each subgroup.

### Statistical analysis

For data analysis, subjects will be assigned into groups according to their diagnosis at the 6-month follow-up. For the primary objective, data from subjects with a diagnosis of MS, AQP4-IgG+ NMOSD, and MOGAD will be analyzed. Data from subjects with other diagnoses (other autoimmune, e.g., CIS-ON, seronegative non-MS-ON) will be described and characterized separately but not included in the primary analysis.

#### Primary objective

We will estimate the treatment effect from a mixed model with visual acuity as an outcome variable, and the following fixed and random factors will be adjusted to the baseline:

- Diagnosis (fixed; 3 levels)
- Days from visual loss (fixed; 3 levels: 0–3 days, 4–7 days, and 8–30 days until high-dose corticosteroid treatment)
- Dosage (fixed)
- Interaction between diagnosis and days since the visual loss (fixed)
- Eye (random; cluster effect to account for dependencies)
- Initiation of disease-modifying treatment (fixed; 2 levels)
- Center (random).

In case of interactions, diagnose-specific effects will be estimated from mixed models along with 95% confidence intervals.

#### Secondary objectives

We will characterize all secondary endpoints descriptively. All secondary methods will be analyzed with standard methods (e.g., *t*-test, Wilcoxon Mann–Whitney test, chi-square test, etc.), depending on their scales (metric vs. non-metric data). All secondary objectives are considered exploratory with limited inferential value. We will apply prediction modeling with measures of performance (accuracy/calibration) to study the diagnostic or prognostic value of novel markers for etiology determination and outcomes.

#### Potential bias and methods to reduce bias

Since this study is observational and thus randomization and masking to treatment assignment are not possible, it has potential sources of the known bias. First, the study has a selection bias, as some people do not present in the clinic for an inaugural ON at all or are more likely to present with a more severe ON. To mitigate this, a patient's medical history is assessed in a semi-standardized way to detect potential previous attacks, leading to study exclusion. In addition, an ophthalmological examination is conducted to assess for evidence of prior ON damage. Furthermore, prior polling of participating centers carried out in 2021/2022 regarding the number of patients with acute ON seen in each clinic demonstrates the feasibility of including a representative population of subjects with inaugural ON of all severities.

Second, the study has a confounder bias, as subjects will receive treatment according to their time of presentation at the hospital as the standard of care. Furthermore, disease-modifying treatments and other medication initiated between the baseline and follow-up will be analyzed as potential confounders. While high-dose corticosteroid treatment cannot be delayed for ethical reasons, a thorough record will keep the process transparent, and confounders will be minimized by advanced biostatistical techniques, such as confounder adjustment.

Information bias may be introduced when recording the primary visual outcomes, stemming from differences in visual acuity recording techniques, room lighting, and different physicians measuring visual acuity. Therefore, a training course for all participating centers will be held, which aims to standardize assessments across centers. To overcome analytic differences within different OCT machines, we have developed a pipeline for device-independent intraretinal layer OCT segmentation (74), allowing for standardized analysis regarding the region of interest and layer boundaries. Additional potential biases include OCT-captured artifacts for which ACON agreed to adhere to the APOSTEL guidelines for performing and reporting OCT measurements (75). Similarly, different MRI techniques pose another source of information bias. To counteract this, ACON will offer short MRI evaluation training to the participating centers, focusing on the interpretation of optic nerve abnormalities.

To overcome biases through assay differences, blood and CSF samples will be stored to perform centralized testing during follow-up.

## Discussion

ACON is the first global prospective longitudinal study on acute ON that includes participating centers from Africa, America, Asia, Australia, and Europe. It intends to build up a comprehensive, systematic, multimodal database of ON patients both in the acute phase and during longitudinal follow-up. Since the publication of the ONTT results in 1992 (21), no large-scale international multicenter study has investigated the treatment of acute ON. In particular, the effect of time to treatment on visual outcomes has not been examined prospectively, either in a multi-racial cohort or with regard to different ON etiologies. Therefore, clinical treatment decisions are largely based on individual choices but lack scientific evidence. ACON will address the question of the potential benefit of hyperacute high-dose corticosteroids on visual acuity, visual function in daily routine, QoL, and optic nerve structural outcomes in acute MS-ON, AQP4-IgG+ON, and MOG-IgG+ON. ACON will investigate the viability of early clinical clues as potential indicators, both for the underlying diagnosis and the respective disease course. The medical history is the first step in the diagnostic workup. To investigate pain scores as a means of distinguishing MS-ON and AQP4-IgG+ON from MOG-IgG+ON, we will carry out a semi-structured interview to assess the chronology of visual symptoms and characterize pain patterns. Data obtained from this interview, collected from multiple countries, including a wide range of races, are expected to result in an easily accessible clinical tool to accelerate the diagnosis with regard to etiology. Furthermore, ACON aims to explore the implications of early dynamics in levels of NfL and GFAP in the serum of patients with acute ON and their potential prognostic value for a subsequent disease conversion to MS, NMOSD, and MOGAD. MRI of the brain and the spinal cord plays an essential role in the diagnosis of these three diseases. For example, prior studies could show that the combination of two radiologic parameters (e.g., absence of brain abnormalities and a greater lesion extension) offers a valuable tool to discriminate between MS-ON and antibody-associated ON (28, 76). Furthermore, a recent study shows that the length of optic nerve inflammation seen in MRI correlates with retinal neuro-axonal loss and chronic visual impairment (29).

ACON will have the potential to provide information about the interrelation between MRI-based lesion characteristics and clinical symptoms, the duration of contrast enhancement, and predictors of the respective diagnosis.

OCT is the method of choice to measure precisely the thickness of retinal layers and to detect structural damage (77, 78). Axonal degeneration begins on a molecular level, within hours of ON onset and can be reliably quantified by OCT after ~3 months (6, 79, 80). OCT-derived retinal measurements have been used as structural biomarkers for disease progression and tissue damage in MS and related disorders (74, 81–88). Thinning of the combined ganglion cell–inner plexiform layer (GCIPL) in non-ON eyes of people with CIS and early MS is associated with future MS disease activity (84, 85). However, there is no such investigation in acute ON. Prior studies from our groups have shown that cumulative axonal damage, macular GCIPL thinning, and visual loss are typically more severe in AQP4-IgG+ON than in MS-ON and MOG-IgG+ON (89–91), and higher grade pRNFL swelling is closely correlated with MOG-IgG+ON in distinction to MS-ON (30).

ACON will investigate the ability to predict the development of clinically manifested MS through acute OCT parameters including pRNFL and macular ganglion cell analysis, as well as advanced parameters such as the shape of the optic nerve head (92).

ACON provides a global effort for collecting real-world information and high-quality prospective multimodal data on subjects with acute ON from 28 participating centers across six continents. It focuses on the longitudinal observation of subjects with MS-ON, AQP4-IgG+ON, and MOG-IgG+ON. With a better understanding of these distinct neuroimmunological conditions, ACON aims to accelerate ON diagnosis and establish acute ON treatment standards that are applicable globally.

Moreover, the ACON study will provide invaluable insights into the course of these diseases. Particularly, ACON has the capacity to give novel insights into MOGAD for which the proportion of monophasic vs. relapsing cases is far from clear (8). Given a follow-up of 12 months minimum (a longer observation period is envisioned), the study will also generate data on the implementation of preventative immunotherapy (when to treat, whom to treat, and potentially which drug to use?). While there is broad consensus that immediate immunotherapy after diagnosis is indicated in AQP4-IgG+NMOSD given the high risk of recurrence and poor prognosis if left untreated (93–95), the situation is less clear in MS and MOGAD (96, 97), and finally, in light of the rapidly changing treatment landscape with approved drugs for AQP4-IgG+NMOSD and several clinical trials in MOGAD currently underway, the ACON study will collect clinically useful data on individual treatment sequences for on-label or off-label immunotherapies in patients with acute ON (97–100).

## Ethics statement

The study was approved by the Ethics Committees from the initiating centers Charité-Universitätsmedizin Berlin (EA2/215/21) and the Rabin Medical Center (0721-18). Ethics Committee approvals and exemptions are obtained separately by each participating center.

## Author contributions

SA, NA, JC, FK, IL, SMa, SR, FP, and HS-K: conceptualization. SA, NA, JB, OB, YB, FB, AC-C, EC, SC, JC, CC, MC, RD, JD, KF, EF, CF, CG-A, JH, MH, HK, PK, FK, CLM, ML-P, MLei, NL, MLev, SL, PL, IL, AL, SMo, RM, SMa, CO, FC, MO, JPa, LP, JPe, SP, SR, NR, AS, SSa, BS-D, DS, MS, TS-H, JS, SSi, PSp, PSu, AT, AV-D, AM, AW-Y, LZ, HZ, FP, and HS-K: investigation. SA, NA, JC, FK, RM, SM, ML, IL, MO, JPa, SR, AW-Y, HZ, FP, and HS-K: methodology. SA, FP, and HS-K: project administration. SA, KF, MO, and AT: software. SA, FP, and HS-K: supervision. SA, FK, PSp, FP, and HS-K: validation. SA and HS-K: visualization and writing—original draft preparation. SA, NA, JB, OB, YB, FB, AC-C, EC, SC, JC, CC, MC, RD, JD, KF, EF, CF, CG-A, JH, MH, HK, PK, FK, CLM, ML-P, MLei, NL, MLev, SL, PL, IL, AL, SMo, RM, SM, CO, FC, MO, JPa, LP, JPe, SP, SR, NR, AS, SSa, BS-D, DS, MS, TS-H, JS, SSi, PSp, PSu, AT, AV-D, AM, AW-Y, LZ, HZ, FP, and HS-K: writing—review and editing. All authors contributed to the article and approved the submitted version.
