# Supplementary material for: The Acute Optic Neuritis Network (ACON): Study protocol of a non-interventional prospective multicenter study on diagnosis and treatment of acute optic neuritis
Source: Front Neurol. 2023 Feb 24;14:1102353. doi: 10.3389/fneur.2023.1102353 (PMC9998999; doi:10.3389/fneur.2023.1102353)
Supplement: Supplementary file 1 [file Data_Sheet_1.docx]

**Supporting information**

Participating centers

| **Africa** | University of Botswana, Botswana |
| --- | --- |
|  | University Teaching Hospital in Lusaka, Zambia |
| **Asia** | Nitte University, Karnataka, India |
|  | Research Institute and Hospital of National Cancer Center, Goyang Korea |
|  | Faculty of Medicine Siriraj Hospital; Bangkok, Thailand |
|  | Hanoi Medical University, Vietnam |
| **Middle East** | Hadassah Medical Center, Jerusalem, Israel |
|  | Sackler School of Medicine and Rabin Medical Center, Tel Aviv, Israel |
| **North America** | Harvard Medical School, Boston, Massachusetts, USA |
|  | Mayo Clinic, Rochester, Minnesota, USA |
|  | University of Colorado School of Medicine, Aurora, Colorado, USA |
| **South America** | Hospital Aléman, Buenos Aires, Argentina |
|  | University of Minas Gerais, State of Minas Gerais, Brazil |
|  | Del Rosario University, Bogota, Colombia |
|  | Fundación Universitaria Sanitas Facultad de Medicina, Bogota, Colombia |
|  | Pontificia Universidad Javeriana, Bogota, Colombia |
| **Australia** | Faculty of Medicine and Health, Sydney, Australia |
| **Europe** | University of Southern Denmark, Odense, Denmark |
|  | Lyon University Hospital, France |
|  | Charité University medicine of Berlin, Germany |
|  | Ludwig-Maximilians-University Munich, Germany |
|  | University Hospitals of Birmingham, United Kingdom |
|  | University of Oxford, United Kingdom |
|  | University of Bologna, Italy |
|  | University of Verona, Italy |
|  | University of Barcelona, Spain |
|  | University Autonoma of Barcelona, Spain |

**S1 Questionnaires and Data Collection CRFsMedical history:**

Acute/current ON

- Which eye is affected by the ON? (Left /Right/Both)
- Did the patient experience/experiences pain or discomfort in or around the affected eye/s? (Y/N)
- When did the pain or discomfort start?
- Did the pain increase with movement of the affected eye/s? (Y/N)
- How intense was the pain on average, ranging from 0 (no pain) to 10 (maximal pain)
- If the pain and/or discomfort was present when the patient received corticosteroids, did they improve with medication? (Dramatically/Somewhat/Not at all/No corticosteroid therapy received)
- On what date did the patient first notice visual symptoms (e.g. visual blur/loss of vision/scotoma)?
- When the visual symptoms started, did the pain or discomfort subside? (If pain occurred bevor visual symptoms) (Y/N)
- Does the patient currently experience eye pain? (Y/N)
- How much time passed from onset of vision loss to NADIR (days)?
- When did the patient last have an infection? (1-3 months/4-6 months/>6 months)
- Date of the last vaccination? (1-3 months/4-6 months/>6 months)
- Did the patient receive a vaccination against SARS-CoV-2? (Y/N)
- If yes, when was the patients’ first/second/third vaccination and what was the type of vaccine did the patient receive?

Fertility anamnesis (for female participants)

- Does the patient have a regular menstrual cycle? (Y/N)
- Does any of the following apply? (Amenorrhoea/Menorrhagia/Frequent intermenstrual bleeding/Menopause/Post-menopause/None applicable)
- Could the patient be pregnant?
- How many pregnancies did the patient have?
- How many children has the patient given birth to? (Please provide dates of birth)

Relapse/Attack history

- Onset of symptoms (date)
- Clinical symptoms
- Affected systems (Visual functions/Brainstem functions/Pyramidial functions/Cerebellar functions/Sensory functions/Bowel and bladder functions/Cerebral functions/Not classifiable)
- Syndromes (Wingerchuck 2015) (Optic neuritis/Myelitis/Area postrema/Brainstem/Diencephalic syndrome/Cerebral syndrome/Not classifiable)
- If optic neuritis, which eye? (Right/Left/Bilateral)
- If myelitis, longitudinally extensive, covering 3 or more spinal segments? (Y/N/unclear)
- Was an MRI done during the attack/relapse? (Yes, without contrast medium/Yes, with contrast medium/No)
- Are contrast enhancing lesions visible? (Y/N)
- Did the patient receive therapy? (Y/N)
- If yes, type of therapy (High-dose corticosteroids i.v./High-dose corticosteroids oral/Plasmapheresis/Immunoadsorption/IVIGs)
- Date, doses and length of treatment
- Was treatment escalated? (Y/N)
- If yes, how? (High-dose corticosteroids i.v./High-dose corticosteroids oral/Plasmapheresis/Immunoadsorption/IVIGs)
- Did the patient receive an oral taper of corticosteroids? (Y/N)
- If yes, duration of taper and total dose

**Comorbidities and regular medication**

- Diagnosed comorbidities, with date of diagnosis and whether still ongoing
- List of all regular medication and supplements, with starting dates, dose, frequency, and application

**ON-associated headache**

- Has the patient experienced migraines? (Y/N)
- How frequently does the patient experience migraines? (Less than 1 per year/1-6 per year/7-12 per year/1-15 per month/More than 15 a month)
- Did the patient experience a headache in the context of the optic neuritis? (Y/N)
- Was this headache more intense than or different to a usual headache? (Y/N)
- The headache began… (Before onset of vision loss/At the same time/After onset of vision loss)
- What was the time gap between the onset of the headache and the onset of vision loss? (Less than 1 hour/1-4 hours/4 hours to 1 day/Several days, up to a week/More than a week)
- If headaches preceded vision loss, did the pain subside when visual symptoms started? (Y/N)
- Where was this headache located? Multiple answers possible (Frontal/Temporal/Parietal/Occipital/Holocephalic/Other (please specify))
- On which side was this headache located? (Right/Left/Bilateral)
- How long did the headache last? (Less than one hour/1-4 hours/4 hours to one day/Several days, up to a week/More than 1 week)
- How intense was the pain on average, ranging from 0 (no pain) to 10 (maximal pain)?
- How would the patient describe the headache? (Pulsating /Dull or tightening /Tingling or stabbing)
- Did the patient suffer from any additional symptoms? (Nausea/Vomiting/Hypersensitivity to light/Hypersensitivity to noise/Pain with eye movement)
- Did usual pain killers relieve the headache? (Yes, completely/Yes, somewhat/No/Not used)
- If the headache was present when the patient received corticosteroids, did it improve? (Dramatically/Somewhat/Not at all/No corticosteroid therapy received)
- Is the patient currently still experiencing headaches? (Y/N)

**Ophthalmological examination**

**Visual acuity**

- Right eye best corrected visual acuity (BCVA) measurement immediately before high-dose corticosteroid therapy
- Left eye best corrected visual acuity (BCVA) measurement immediately before high-dose corticosteroid therapy
- Distance high contrast letter acuity (HCLA) vision testing (in meters)
- Right Eye: 100% HCLA
- Left Eye: 100% HCLA
- Swollen disc visible during fundoscopy (Y/N)
- Signs of peripapillary/retinal haemorrhage (Y/N)
- Description of scotoma & visual field defects (from a set of descriptions) plus mean deviation detected on perimetry for right eye
- Description of scotoma & visual field defects (from a set of descriptions) plus mean deviation detected on perimetry for left eye

**Visual Evoked Potentials**

- Right eye LATENCY (P100 msec average (avg.) of 5-10 readings)
- Right eye LATENCY (P100 msec avg. of 5-10 readings)

**Optical Coherence Tomography**

- Right eye signal strength (5-10), do not include if < 5
- Left eye signal strength (5-10), do not include if < 5
- Right eye DISC AREA
- Left eye DISC AREA
- Right eye avg. retinal nerve fiber layer (RNFL) (microns)
- Left eye avg. pRNFL (microns)
- Right eye avg. Ganglion cell layer (GCL)+inner plexiform layer (IPL) (microns)
- Left eye avg. GCL+IPL (microns)
- Right eye avg. Disc Cupping (no. 0.0-1.0)
- Left eye avg. Disc Cupping (no. 0.0-1.0)
- Right eye vertical Disc Cupping (no. 0.0-1.0)
- Left eye vertical Disc Cupping (no. 0.0-1.0)

**Blood and cerebrospinal fluid (CSF)**

- Blood: routine tests (complete blood count, electrolytes); vitamin B12, vitamin D, AQP4-antibody positivity/ negativity, AQP4-antibody ratio, MOG-antibody positivity/negativity, MOG-antibody ratio.
- CSF (only at disease onset): cell count, erythrocytes, lactate, total protein, positivity/negativity of oligoclonal bands.

**Magnet resonance imaging (MRI)**

- Presence of brainstem lesions
- Presence of brain lesions
- Brain/Brainstem T2 lesion number (total number)
- Brain/Brainstem T2 lesion volume (total volume in cm^3^)
- Brain/Brainstem total number of T1 contrast-enhancing lesions
- Brain/Brainstem total volume of T1 contrast-enhancing lesions
- Localization of supratentorial lesions
- Cortical lesions
- Juxtacortical lesions
- Lesions ranging over cortical AND juxtacortical regions
- Lesions ranging over juxtacortical AND periventricular regions
- Periventricular lesions adjacent to the lateral ventricles
- Periventricular lesions including corticospinal tract/s
- Subcortical deep white matter lesions
- Subcortical deep white matter lesions including the corpus callosum
- Diencephalic lesions
- Diencephalic lesions including the thalamus
- Diencephalic lesions including the hypothalamus
- Diencephalic lesions surrounding the third ventricle/cerebral aqueduct
- Localization of infratentorial lesions
- Cerebellar lesions
- Mesencephalon lesions
- Pons lesions
- Medulla oblongata lesions
- Dorsal medula oblongata/area postrema lesions
- Superior cerebellar peduncle/s lesions
- Middle cerebellar peduncle/s lesions
- Peri ependymal brainstem lesions (surrounding the fourth ventricle)
- Presence of leptomeningeal enhancement
- Presence of MRI optic nerve abnormalities (including T2 hyperintensity, optic nerve swelling, optic head swelling, and/or contrast enhancement)
- Presence of optic nerve T2 hyperintensities
- Severity of optic nerve T2 hyperintensities
- Presence of optic nerve contrast enhancement
- Severity of contrast enhancement
- Presence of optic nerve edema (when compared to the contralateral unaffected) optic nerve in unilateral ON, or an ipsilateral unaffected segment of optic nerve in bilateral ON
- Inflammatory extension to orbital fat
- Optic nerve head swelling (that is optic nerve head elevation)
- Most affected optic nerve segment (Retrobulbar/Canalicular/Intracranial)
- Severity of optic nerve involvement (None/Focal/Longitudinal, defined as involving two or more contiguous optic nerve segments, with segments comprising anterior orbital, posterior orbital, canalicular, or intracranial)
- Lesion length in the maximally affected eye, measured in millimeters (mm) (calculated by multiplying the slice thickness plus inter-slice interval by the number of coronal sections through which the optic nerve changes were visible (total volume (mm^3^))
- Involvement of optic chiasm
- Involvement of optic tract
- Involved segments of the visual pathway divided into 12 segments (anterior orbital, posterior orbital, canalicular, intracranial, chiasmal, and tract segments for each of the left and right optic pathways)
- Lesion sum score (sum of involved segments, 6 segments per eye) (x/12)
